# Genome-wide identification of the *NAC* gene family and its functional analysis in *Liriodendron*

**DOI:** 10.1186/s12870-023-04415-4

**Published:** 2023-09-08

**Authors:** Siqin Liu, Yuanlin Guan, Yuhao Weng, Bojun Liao, Lu Tong, Zhaodong Hao, Jinhui Chen, Jisen Shi, Tielong Cheng

**Affiliations:** 1 State Key Laboratory of Tree Genetics and Breeding, Co-Innovation Center for Sustainable Forestry in Southern China, Nanjing Forestry University, Longpan Road 159, Nanjing, 210037 China; 2 Key Laboratory of Forest Genetics and Biotechnology of Ministry of Education, Nanjing Forestry University, Nanjing, 210037 China; 3 College of Biology and the Environment, Nanjing Forestry University, Nanjing, 210037 China

**Keywords:** *Liriodendron chinense*, NAC transcription factor, Expression pattern, Somatic embryo development, Abiotic stress

## Abstract

As one of the largest plant specific transcription factor families, NAC family members play an important role in plant growth, development and stress resistance. To investigate the function of NAC transcription factors during abiotic stress, as well as during somatic embryogenesis, we identified and characterized the *NAC* gene family in *Liriodendron chinense*. We found that most *LcNAC* members contain more than three exons, with a relatively conserved gene and motif structure, especially at the N-terminus. Interspecies collinearity analysis revealed a closer relationship between the *L. chinense* NACs and the *P. trichocarpa* NACs. We analyzed the expression of *LcNAC* in different tissues and under three abiotic stresses. We found that 12 genes were highly expressed during the ES3 and ES4 stages of somatic embryos, suggesting that they are involved in the development of somatic embryos. 6 *LcNAC* genes are highly expressed in flower organs. The expression pattern analysis of *LcNACs* based on transcriptome data and RT-qPCR obtained from *L. chinense* leaves indicated differential expression responses to drought, cold, and heat stress. Genes in the NAM subfamily expressed differently during abiotic stress, and *LcNAC6/18/41/65* might be the key genes in response to abiotic stress. *LcNAC6/18/41/65* were cloned and transiently transformed into *Liriodendron* protoplasts, where *LcNAC18/65* was localized in cytoplasm and nucleus, and *LcNAC6/41* was localized only in nucleus. Overall, our findings suggest a role of the *NAC* gene family during environmental stresses in *L. chinense*. This research provides a basis for further study of *NAC* genes in *Liriodendron chinense*.

**Supplementary Information:**

The online version contains supplementary material available at 10.1186/s12870-023-04415-4.

## Background

Transcription factors (TFs) are a class of proteins that regulate the expression of target genes by binding to the specific *cis*-acting elements of their promoters. Plant TFs such as AP2, bHLH, ARF, MYC, WRKY and NAC, are essential regulators in many biological processes [1–4]. Among them, the *NAC* gene family is one of the largest families of transcription factors in plants. The *NAC* gene family name is composed of the initials of NAM (*No Apical Meristem* from *Petunia hybrida*), AF1/2 (*transcriptional activator 1/2* from *Arabidopsis thaliana*) and CUC2 (*cup-shaped cotyledon from Arabidopsis thaliana*) transcription factors. So far, the *NAC* gene family has been characterized in a large number of plant species, including 117 *NAC* genes in *Arabidopsis thaliana*, 151 *NAC* genes in *Oryza sativa* L. subsp. *japonica cv.* [5], 72 *NAC* genes in perennial ryegrass (*Lolium perenne* L.) [6], 93 *NAC* genes in tomato (*Solanum lycopersicum*) [7], 73 *NAC* genes in Pineapple (*Ananas comosus*) [8], 180 *NAC* genes in apple (*Malus domestica*) [9] and 170 *NAC* genes in *Populus trichocarpa* [10].

NAC transcription factors usually have a highly conserved DNA-binding domain, a NAC domain, situated at the N-terminus, and a variable transcriptional regulatory region located at the C-terminus of the protein [11]. Generally, the N-terminal NAC domain is about 150 amino acid residues in length and consists of five (A-E) subdomains. The highly conserved subdomains C and D are predicted to bind to DNA, subdomain A is involved in homo- and heterodimerization, while the divergent subdomains B and E influence the functional diversity of NAC proteins [12, 13].

Many studies have shown that *NAC* genes play important roles in various biological processes, including in the regulation of flower and leaf development, secondary cell wall thickening, protein and lipid metabolism pathways, leaf senescence and fruit development, lateral root formation and seed germination. For example, in the *cuc1*; *cuc2* double mutant of *Arabidopsis thaliana*, sepals, stamens and cotyledons fuse and apical meristem tissue has difficulty to form [14]. *RhNAC100* in *Rosa hybrida* is post-transcriptionally regulated by ethylene through miR164, and overexpression of *RhNAC100* significantly reduces petal size by inhibiting petal cell expansion [15]. In addition to the involvement of *NAC* in plant growth, some studies have shown that *NAC* family members play a role in somatic cell development. In the study of *Dendrobium candidum*, zhao et al. analyzed the RT-PCR and in situ hybridization of *DcNAC* and found that *DcNAC* was involved in the development and maturation of embryos [16]. Larsson et al. found that *PaNAC01* of *Picea abies* was cloned into a *cuc1cuc2* double mutant of *A. thaliana*, where *PaNAC01* could functionally replace *CUC2* and was associated with SAM differentiation and cotyledon formation [17]. Munir et al. identified *Dimocarpus longan* Lour and explored expression patterns during early somatic embryogenesis, and found that the expression of *DlNACs* at EC and GE stages was higher than that at ICpEC stages [18].

More and more reports have shown that NAC proteins play a major role in abiotic stress response [19]. The grape transcription factor *VvNAC17*, when overexpressed in *A. thaliana*, increases sensitivity to abscisic acid and improves salt, freezing and drought tolerance of the transgenic plant [20]. Overexpression of the *SlNAC35* transcription factor improves cold tolerance of transgenic tomato plants [21]. The stress-responsive transcription factor *SNAC3*, when overexpressed in rice, increases heat and drought tolerance by regulating reactive oxygen species [22]. The transcription factors *ANAC096* and *bZIP* act cooperatively in regulating dehydration and osmotic stress response in *A. thaliana* [23]. Overexpression of *VvNAC08* from grapevine enhances drought tolerance in *A. thaliana* [24]. *TaNAC29* from wheat, when overexpressed in *A. thaliana*, enhances its salt and drought tolerance [25]. Plants with ectopic expression of *MlNAC12* have a higher rate of survival and experience reduced water loss, resulting in increased drought resistance [26].

*L. chinense* (*Liriodendron chinense* (Hemsl.) Sarg.) is a deciduous tree from the Magnoliaceous family, a rare species in China that often grows in mountainous forested areas at an altitude of 1000m [27]. Its flowers are shaped like tulips and its wood has a high commercial value. However, unfavorable environmental conditions such as heat, cold and drought affect the growth and development of *L. chinense* [28, 29]. As mentioned above, *NAC* transcription factors play an important role in response to abiotic stress, but *NAC* genes have rarely been studied in *L. chinense*. To investigate how *L. chinense* responds to abiotic stress, we focused on its endogenous *NAC* gene family. In this study, we performed genome wide identification of the *NAC* gene family members in *L. chinense*. We studied their subcellular localization, phylogenetic relationships and conserved motifs, and analyzed transcriptome data to study the expression of *NAC* genes at different developmental stages. This research provides a basis for further exploration of the function of *NAC* genes in *L. chinense*.

## Material and methods

### *NAC* gene identification

The complete genome, protein sequences, and associated files of *L. chinense* were downloaded from NCBI (https://www.ncbi.nlm.nih.gov/assembly/GCA_003013855.2). The *A. thaliana* genome data was downloaded from the TAIR (https://www.arabidopsis.org/) database. The NAM domain (PF02365) hidden Markov model file was retrieved from the Pfam website (https://pfam.xfam.org/). The NAM domain was utilized to search for LcNAC protein sequences using HMMER3.0 software with an E-value threshold of 1E-3. All candidate LcNAC proteins were aligned against AtNACs with BLASTp (v2.9.0) under an E-value cutoff of 1E-5. Finally, all candidate *LcNAC* genes were validated using the Conserved Domain Search tool (https://www.ncbi.nlm.nih.gov/Structure/cdd/wrpsb.cgi). The online website (http://web.expasy.org/Compute-pI/) was utilized for the prediction of molecular weight (MW) and isoelectric point (pI). The conserved NAC motifs of *L. chinense* were subjected to analysis using the MEME tool (https://meme-suite.org/meme/). *Cis*-acting elements of *LcNAC* genes were analyzed using Plantcare (http://bioinformatics.psb.ugent.be/webtools/plantcare/html/).

### Phylogenetic tree construction, chromosomal localization, collinearity analysis and protein interaction network prediction

The NAC protein sequences of *A. thaliana* and *L. chinense* were analyzed using Jalview software. The phylogenetic tree was constructed using MEGA7with the maximum likelihood method and 1000 bootstrap replicates [30]. We mapped the *LcNAC* gene chromosomal positions using TBtools software (TBtools.v1.09854). The collinearity and gene replication analysis were performed with the Dual Systeny Plot for MCscanX, Circle Gene View and Advanced Circos in TBtools. *L. chinense* NAC proteins were compared to their *A. thaliana* homologues using Blastp, referring to the known network relationship of *A. thaliana* NAC proteins on the String website (https://string-db.org/). The interaction of NAC proteins in *L. chinense* was predicted, and the NAC protein interaction network of *L. chinense* was constructed, with the default parameter settings.

### Transcriptome sequencing of somatic embryos in* L. chinense*

Embryonic callus of *L. chinense* was induced to form somatic embryos in liquid suspension culture, and samples were collected at successive somatic embryo stages (11 stages) for RAN-seq (data not yet published). Stages were as follows: embryonic callus culture for 20 days (PEMs), liquid suspension culture for 10 days (ES1), single cell culture for 2 days (ES2), ABA induction for 1 day (ES3), ABA induction for 3 days (ES4), 7 days spherical embryo (ES5), 13-day heart-shaped embryo (ES6), 19-day torpedo embryo (ES7), 25-day immature cotyledon embryo (ES8), 31-day mature cotyledon embryo (ES9) and 37-day plantlet (PL). The Fragments Per Kilobase per million mapped fragments (FPKM) data of *NAC* genes at different stages of somatic embryos is shown in Supplementary Table S1.

### Material culture, abiotic stress treatment, RNA-seq and RT-qPCR analysis of *L. chinense*

The conditions in the growth chamber were set as follows: 23 °C, 5000 Lux light intensity, 16 h light/8 h darkness, relative humidity 55% [31]. 36 seedlings with 3–4 true leaves were divided into three groups and subjected to 40℃ for heat treatment, 20% polyethylene glycol (PEG6000) for drought treatment and 4℃ for cold treatment, respectively. Leaf samples were collected at 0 h, 1 h, 3 h, 6 h, 12 h, 24 h and 3d, respectively. Total RNA was isolated and followed by RNA-seq. Raw data was carried out quality control through FastQC (v0.12.1) and then were filtered by using Trimmomatic (v0.39) according to the quality control results, parameters as followed: remove adapters (ILLUMINACLIP: TruSeq3-PE.fa:2:30:10), remove leading low quality or N bases (LEADING:10), remove trailing low quality or N bases (TRAILING:10), scan the read with a 4-base wide sliding window, cutting when the average quality per base drops below 20 (SLIDINGWINDOW:4:20), drop reads below the 90 bases long (MINLEN:90). The clean reads were obtained from the Raw data by this step. The Q20, Q30, and GC content of the clean data were calculated simultaneously. High quality clean data can be used for analysis in the next step.

The paired-end clean reads were aligned to the *Liriodendron* genome that was built using Hisat2 (v2.2.1). Then we used DESeq2 (v1.16.1) to determine differentially expressed genes (DEGs) with the (ANOVA method: p.adjust < 0.05, |Log2FC|> 1). Then we selected the Kallisto (v0.46.2) to quantify gene expression by calculating all clean reads. The Fragments Per Kilobase per million mapped fragments (FPKM) values of individual genes were counted and FPKM values were used to calculate the transcript abundance of *LcNACs*. The transcriptome data can be downloaded from the NCBI (https://www.ncbi.nlm.nih.gov/bioproject/PRJNA679089/) & (https://www.ncbi.nlm.nih.gov/bioproject/PRJNA679101/). The transcriptional abundance of *LcNAC* genes at different time point of stress treatment was calculated based on transcriptome FPKM data, as shown in Supplementary Table S2 (cold and heat: PRJNA679089; drought: PRJNA679101). Phloem and xylem samples were obtained simply by separating the bark/phloem from the xylem core. A heat map was constructed (FPKM + 1, log base 2) using TBtools software [32].

Plant total RNA was extracted according to the KK Fast Plant Total RNA Kit (ZP405K) instructions and cDNA was synthesized using a reverse transcription kit. RT-qPCR-specific primers were designed using Primer (premier 6.0) software on the basis of the reference gene sequence, as shown in Supplementary Table S3. A LightCycler480 II (Roche, Switzerland) was used to detect expression of the gene of interest. SYBR Green Premix Pro Taq HS qPCR Kit (AG11701, ACCURATE BIOTECHNOLOGY, HUNAN, Co., Ltd.) was used for qPCR reactions. The RT-qPCR reaction procedure used was 95 °C for 2 min; 95 °C 5 s, 60 °C 30 s, 40 cycles, each reaction was performed in three biological triplicates and three technical replicates. *LcActin* and *LcUBQ10* were used as dual internal reference genes [31, 33], and the gene expression calculation was analyzed via the 2^−ΔΔCt^ method, visualized by Graphpad software.

### Subcellular localization

*LcNAC6/18/41/65* target fragments were obtained by PCR. The plasmid CaMV35S: GFP in pCAMBIA1302 was digested with NcoI enzyme, and the linear vector fragment was ligated with the target gene fragment to construct the CaMV35S: NACs-GFP fusion expression vector. Plasmids were extracted using an endotoxin-free plasmid extraction kit (DP117, TIANGEN). CaMV35S: NACs-GFP and CaMV35S: H2B-MCherry plasmids were transformed into protoplasts by PEG mediated transient transformation method. After 3 days of dark culture, GFP was observed using a laser confocal microscope (Zeiss LSM800).

## Results

### Identification and phylogenetic analysis of the *L. chinense NAC* gene family

**Fig. 1:**
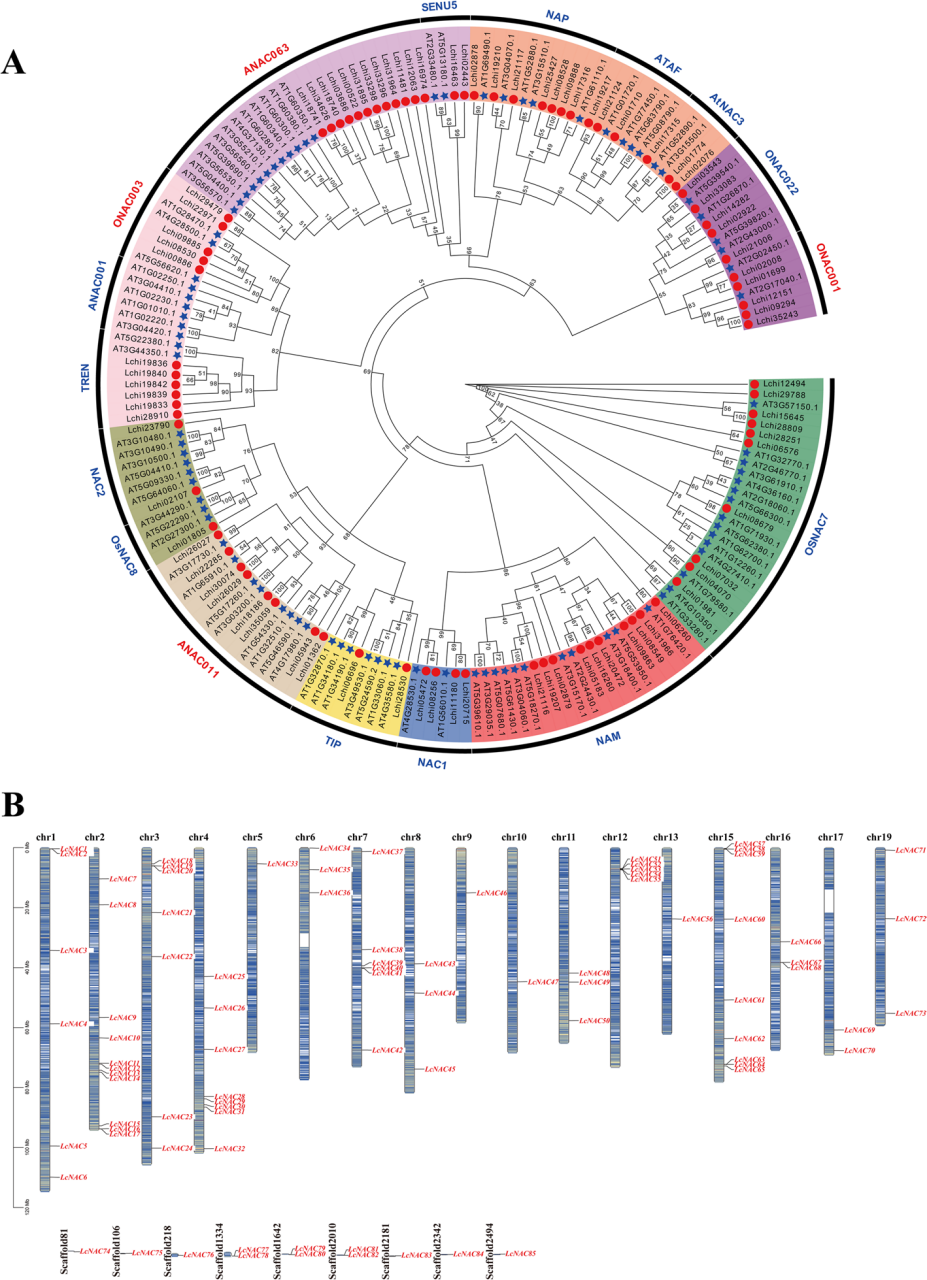
Phylogenetic tree analysis and Chromosomal location of the *NAC* gene family in *L. chinense.* **A** Phylogenetic tree analysis of the *L. chinense* and *A. thaliana NAC* genes. Blue stars represent *A. thaliana* and red circles represent *L. chinense*. **B** Distribution of *LcNAC* genes on *L. chinense* chromosomes and scaffolds. All named *LcNAC* genes are displayed on *L. chinense* chromosome, with the chromosome number marked at the top of each strip. The lines inside the chromosomes represent gene density

The NAM domain was used as input to search for LcNAC protein sequences through HMMER3.0 software. 85 *NAC* gene family members were identified in *L. chinense*, 0.73 times the amount of *NAC* genes in the *A. thaliana* genome. We named the individual genes *LCNAC01-LCNAC85*, based on their position on the chromosome. The *LcNAC* genes are unequally distributed across the 17 *L. chinense* chromosomes, and a few genes could not be pinpointed to a definite chromosomal location, since they were present on genomic scaffolds (Fig. 1B). All LcNAC proteins contain either a conserved NAC domain (PF01849) or a NAM domain (PF02365). We analyzed the physicochemical properties of the 85 *LcNAC* genes, including amino acid number, molecular weight, isoelectric point and predicted subcellular localization (Supplementary Table S4). Amino acid length ranged from 102 (LcNAC36) to 842 (LcNAC66), the relative molecular weight ranged from 11337.81 Da (LcNAC36) to 93486.99 Da (LcNAC66), which is positively correlated with the amino acid number. 42 genes are basic proteins (pI > 7), and others are acidic proteins (pI < 7) among the *NAC* gene family and they have an isoelectric point between 4.19 (LcNAC27) and 9.85 (LcNAC63).

To explore the evolutionary interrelatedness of the *LcNAC* genes, we used the NAC protein sequences from *A. thaliana* and *L. chinense* to construct a phylogenetic tree. *AtNACs* can be subdivided into 17 subgroups [34], while *LcNAC* genes are divided into 16 subgroups (Fig. 1A). The ANAC063 subfamily contains 12 genes, making it the biggest subfamily. The ANAC001 subfamily contains 6 *A. thaliana* genes, but no *LcNAC* genes. The number of *LcNAC* members within each subfamily varies greatly.

### Domain, motif and gene structure analysis of *LcNACs*

**Fig. 2:**
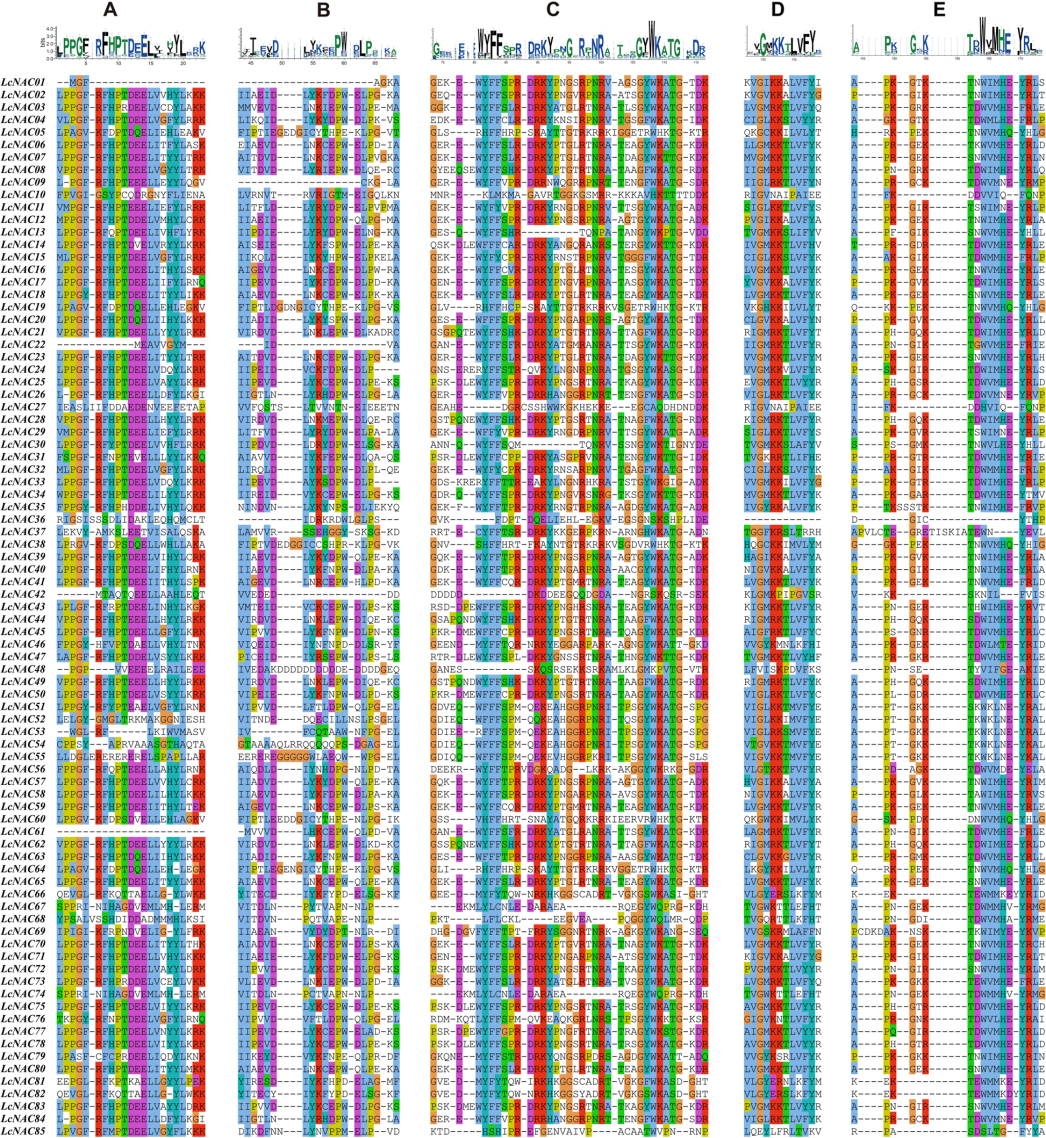
Multiple sequence alignment of the NAC domains from 85 LcNAC proteins. Some of the incomplete sequences or sequences which do not contain N-terminal domains were omitted from the above alignment. No color means that the similarity between amino acid residues of *NAC* gene family members at this site is less than 35%

NAC proteins have a conserved NAM domain for DNA binding which is a key region for the biological functions of NAC proteins. Therefore, we focused on the differences between the domain sequences of *L. chinense* NAC members. We divided the NAC domain of *L. chinense* into five subdomains A-E. Most LcNAC proteins contain five conserved subdomains within their amino terminus (Fig. 2), indicating their strong sequence conservation during the evolutionary process. However, a few genes (*LcNAC01*, *LcNAC22*, *LcNAC42*, *LcNAC61*) have incomplete subdomains.

**Fig. 3:**
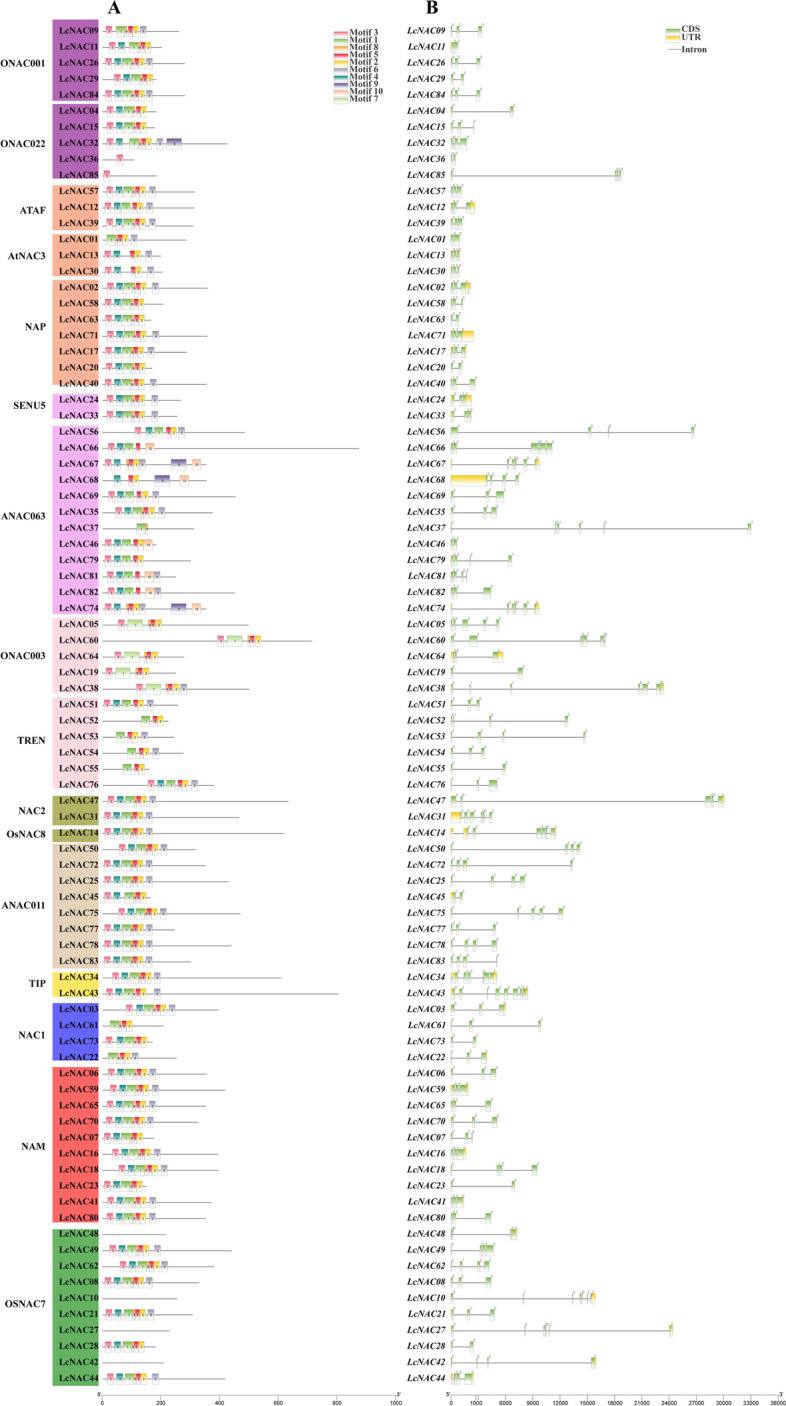
Gene structures of the *L. chinense NAC* gene family. **A** Conserved motifs of LcNAC proteins. **B** Exon–intron structures of *LcNAC* genes. Yellow boxes represent untranslated regions (UTR), and green boxes represent coding sequence (CDS)

To further explore LcNAC protein sequences, we analyzed the conserved motifs of LcNAC proteins. Ten conserved motifs were identified (Fig. 3A), ranging in length from 8 to 50aa (Supplementary Table S5). Motifs 1–5 correspond to the subdomains A-E. Motif 10 is only present in a small number of *LcNAC* genes. Further analysis of its evolutionary origin revealed that the protein sequences with motif 10 belong to subfamily ANAC063. LcNAC36 and LcNAC85 contain only one conserved motif (Motif3). In addition, some NAC family members, such as LcNAC10, LcNAC27, LcNAC42, LcNAC48, do not contain these motifs.

To further understand the structure of *LcNAC* genes, we analyzed their intron/exon compositions (Fig. 3B). The number of introns ranged from 1 to 7 and the number of exons ranged from 2 to 8, with *LcNAC43* containing the largest number of introns (7) and exons (8). Most *LcNAC* genes contain three exons. Genes from the same subfamilies are similar in exon length, intron position and number, indicating that the *NAC* family gene structure is relatively conserved.

### Gene duplication and collinearity analysis of *LcNACs*

**Fig. 4:**
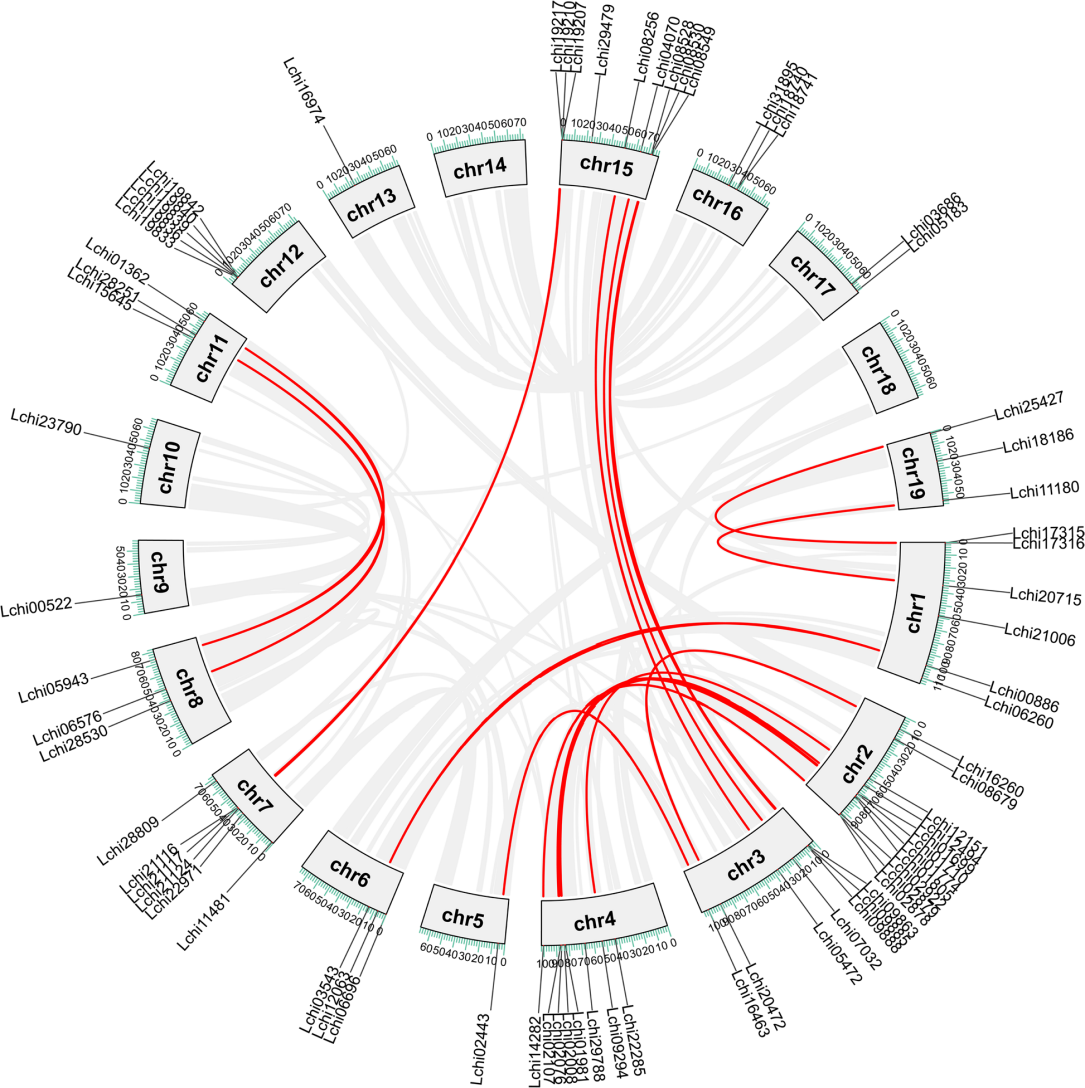
Collinearity of the *L. chinense NAC* gene family*.* Intraspecies collinearity analysis of *LcNAC* genes. The red lines represent duplication events of *LcNAC* genes, the chromosome number is labeled within the gray rectangles

To address to what extent gene duplication has contributed to LcNAC family expansion, we annotated and analyzed the intraspecific collinearity of *LcNAC* genes. The results showed that there are 20 collinearity pairs within the *LcNAC* gene family (Fig. 4). Chr2 and Chr4 contain the highest number of duplicated genes, while the other three paralogous gene pairs are located on Chr3 and Chr15 chromosomes. The remaining chromosomes contain either a single or two segmental duplications.

**Fig. 5:**
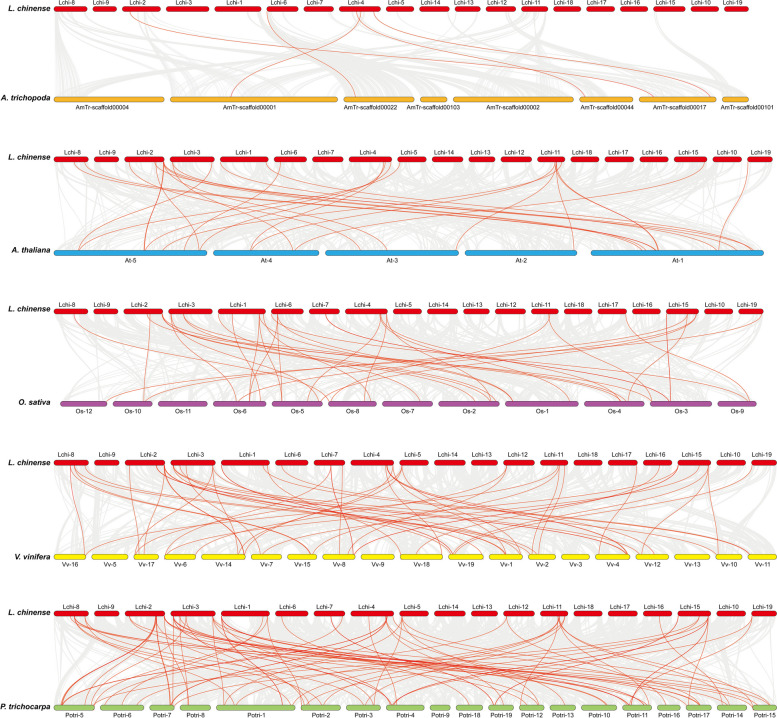
Synteny analysis of *NAC* genes in *L. chinense* with *A. trichopoda*, *A. thaliana*, *O. sativa*, *V. vinifera* and *P. trichocarpa*. Gray lines in the background represent the collinear blocks within *L. chinense* and other species, while the colored lines highlight the collinear *NAC* gene pairs

To further explore the evolutionary interrelationships of the *NAC* genes in different species, we performed interspecies collinearity analysis between *L. chinense* and *A. trichopoda*, *A. thaliana*, *O. sativa*, *V. vinifera* and *P. trichocarpa* (Fig. 5). The *NAC* family members of* L. chinense* have the most collinear pairs with *P. trichocarpa*, with a total of 82 collinear pairs, indicating a close evolutionary relationship between the NAC genes from *L. chinense* and *P. trichocarpa*. Furthermore, *LcNAC14*, *LcNAC28* and *LcNAC50* genes in *L. chinense* have homologous relationships with *A. thaliana*, *V. vinifera*, and *P. trichocarpa*. In addition, there were 28 *NAC* collinear gene pairs between *L. chinense* and *A. thaliana*. The protein sequences were compared between *L. chinense* and* A. thaliana*, revealing that *LcNAC49* is highly homologous with *AT1G32770*, *AT2G46770* and *AT3G61910* genes of* A. thaliana*. *AT3G61910* is specifically expressed in both interfascicular fibers and xylem, and acts as a negative regulator in the thickening process of the secondary wall, suggesting the *LcNAC49* gene may have a similar function in fiber biosynthesis.

### Protein interaction prediction of LcNACs

**Fig. 6:**
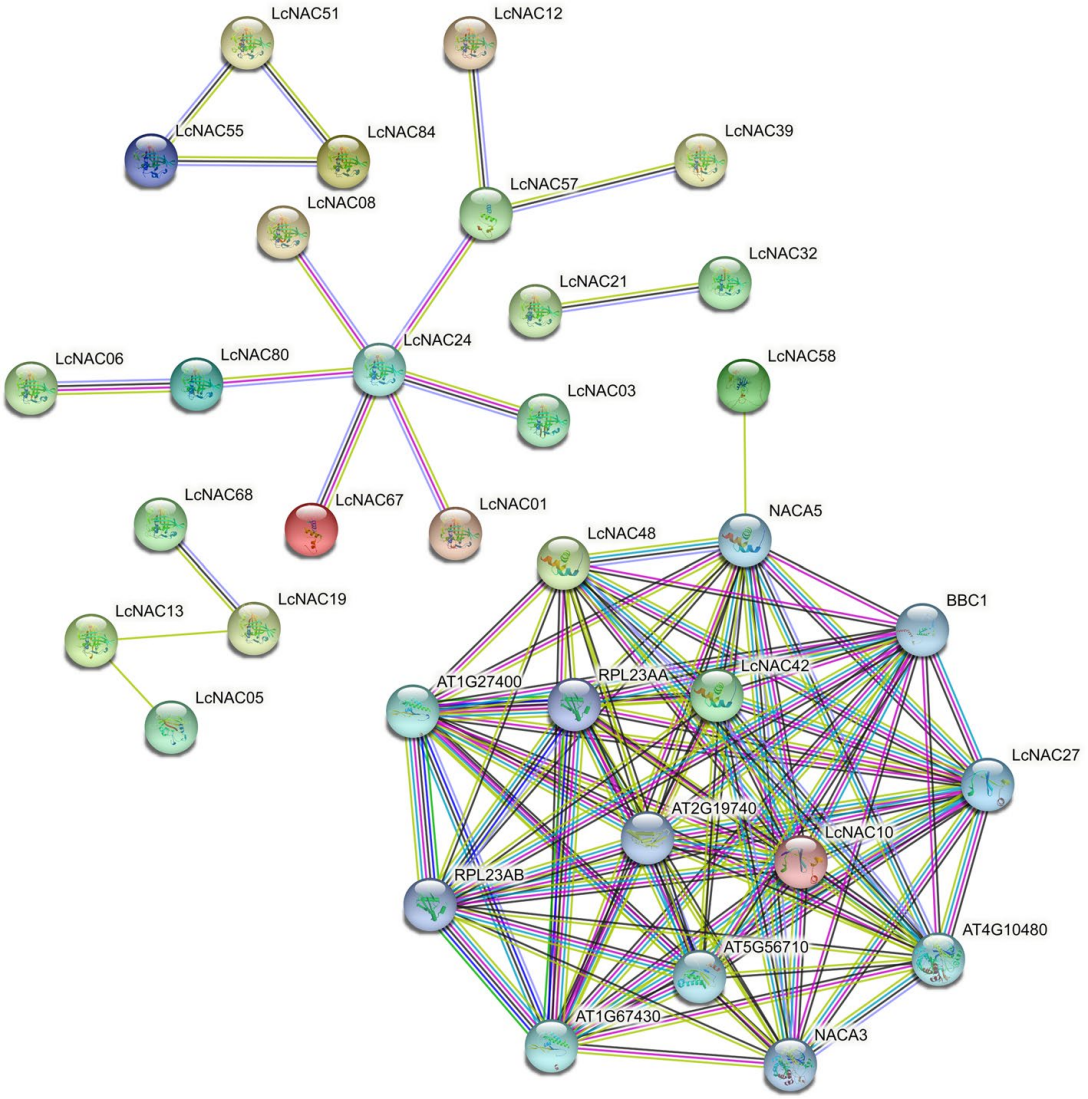
Predicted protein–protein interaction network for the *L. chinense NACs*. Different line colors represent different types of protein–protein interactions. colored nodes: query proteins and first shell of interactors, white nodes: second shell of interactors, filled nodes: some 3D structure is known or predicted

The interactive relationship of the NAC proteins in *L. chinense* was predicted by using the interaction of *A. thaliana* NAC proteins as a reference. We found that a single *A. thaliana* NAC protein corresponded to one or more NAC proteins of *L. chinense*. 9 LcNAC proteins (LcNAC12, LcNAC57, LcNAC24, LcNAC80, LcNAC06, LcNAC01, LcNAC67, LcNAC39 and LcNAC03) may be key nodes in the network (Fig. 6). In addition, there is the interaction between NAC and other transcription factors. It is hypothesized that the regulatory cascade of NAC083-CUC2-CUC3 and NAC083-NAC102-ATAF1/2 may be important to perform their functions, suggesting that NACs may be involved in organ growth, development and stress response.

### Analysis of *LcNACs Cis*-acting elements

**Fig. 7:**
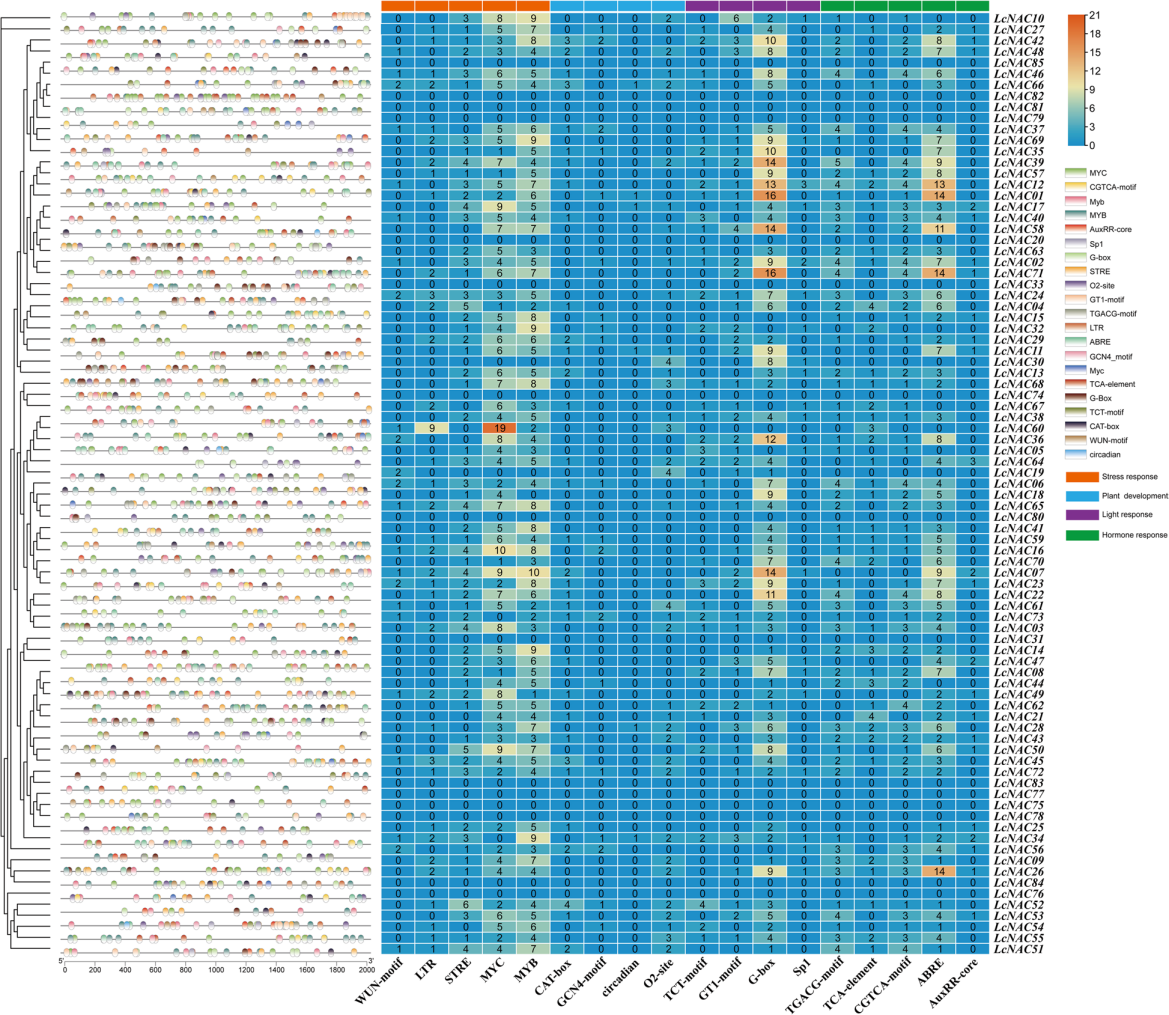
*Cis*-acting elements within the promoter region of *L. chinense NAC* genes. It represents the number of various cis-acting elements in the promoter region of each *LcNAC* gene on the right, and squares with different colors on the left represent different types of cis-acting elements and their positions in the promoter region

To predict the upstream regulators of *LcNAC* genes, we analyzed the 2000 bp upstream of the *LcNAC* genes’ transcription start site through the PlantCARE database (Fig. 7). We identified 310 ABRE elements (abscisic acid response elements) indicating possible ABA-dependent regulation, 63 LTR elements (cold temperature response), 361 MYB binding sites (drought response), 48 CAT-box elements (meristem expression associated) and 30 WUN-motif elements (defense and stress response). In addition, response elements for light, circadian rhythm, auxin and methyl jasmonate were identified. *Cis*-elements are enriched especially in the NAM subfamily. The results suggest that *LcNAC* genes might be involved in environmental stresses, growth regulation and hormonal regulation, and play important roles in physiological and developmental processes.

### Expression patterns of *LcNAC* genes during somatic embryogenesis

**Fig. 8:**
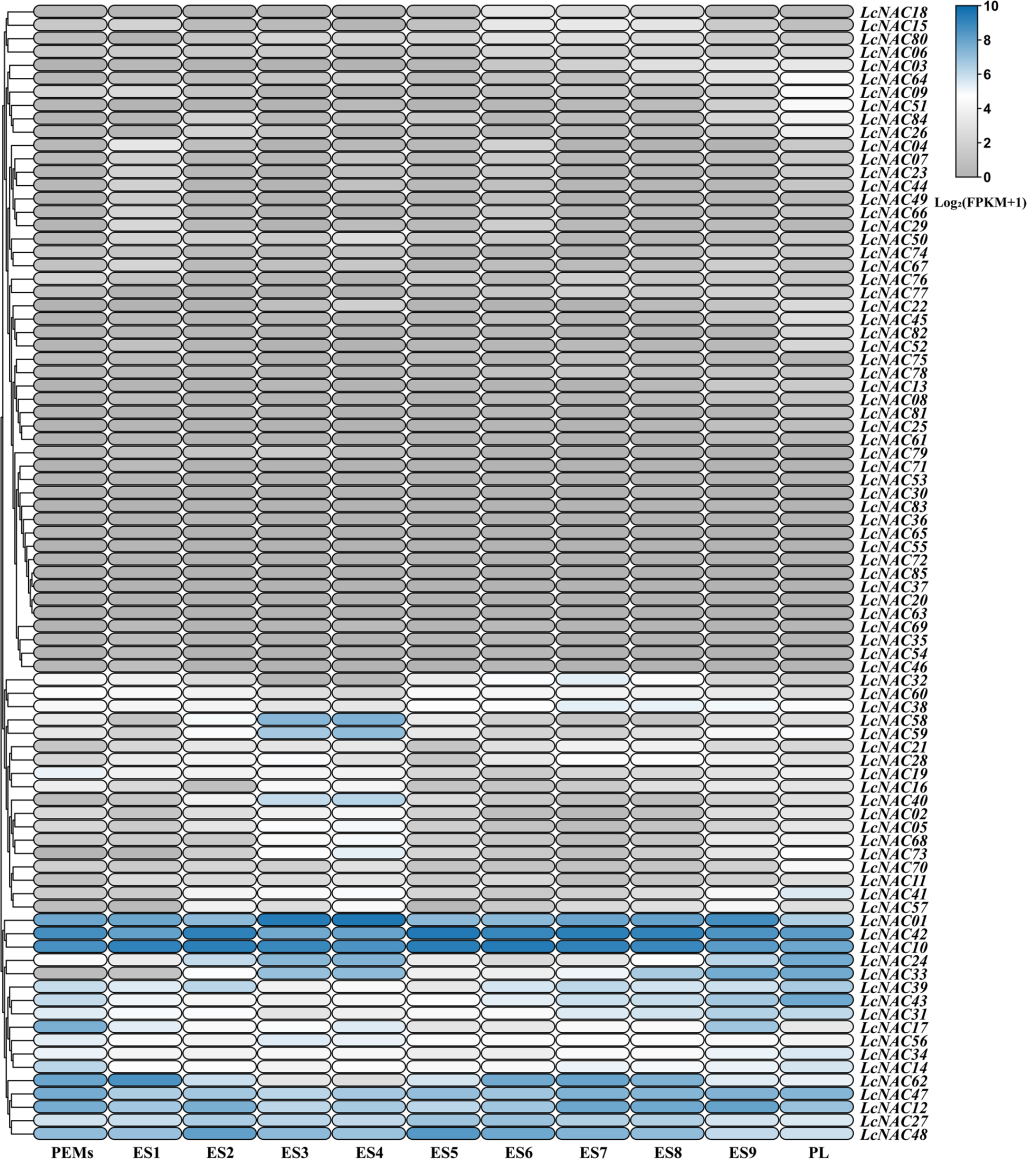
Expression patterns of *NAC* genes in *L. chinense* at different stages of somatic embryonic development. Embryonic callus culture for 20 days (PEMs), liquid suspension culture for 10 days (ES1), single cell culture for 2 days (ES2), ABA induction for 1 day (ES3), ABA induction for 3 days (ES4), 7 days spherical embryo (ES5), 13-day heart-shaped embryo (ES6), 19-day torpedo embryo (ES7), 25-day immature cotyledon embryo (ES8), 31-day mature cotyledon embryo (ES9) and 37-day plantlet (PL). The color key indicates the range of log 2 transformed FPKM + 1 values. The blue represents high expression level and gray represents low expression level

*NAC* genes are involved in somatic embryo development [35]. Consistently, our analysis of *cis*-acting elements in the promoter region of *LcNAC* genes showed that it contains regulatory elements associated with embryogenesis. To explore the specific roles of *NAC* genes during somatic embryogenesis, we analyzed RNA-seq expression data of 11 successive somatic embryo developmental stages (PEMs, ES1, ES2, ES3, ES4, ES5, ES6, ES7, ES8, ES9, PL, see methods) (Fig. 8). More than half of the *LcNAC* genes are not present during somatic embryogenesis. There are three expression patterns in general; 1) some *LcNAC* genes are highly expressed during all 11 stages, such as *LcNAC01*, *LcNAC42*, *LcNAC10*, *LcNAC47*, *LcNAC12*, *LcNAC27* and *LcNAC48*. 2) some *LcNAC* genes are specifically expressed during certain specific stages. For example, the expression level of *LcNAC58*, *LcNAC59* and *LcNAC40* genes during the ES3 and ES4 stage is a hundred times higher than that of PEMs. The *LcNAC41* gene was expressed more than twenty times more strongly in the PL than in the PEMs. 3) some genes are expressed in waves during embryo development; the expression of *LcNAC62* in PEMs-ES1 and ES6-8 stages was very high, but its expression decreased during the remaining stages of embryo development. These results suggest that some *LcNAC* genes are involved in somatic embryo development, especially in regulating certain developmental stages.

### Expression profiling of *LcNAC* genes across different tissues

**Fig. 9:**
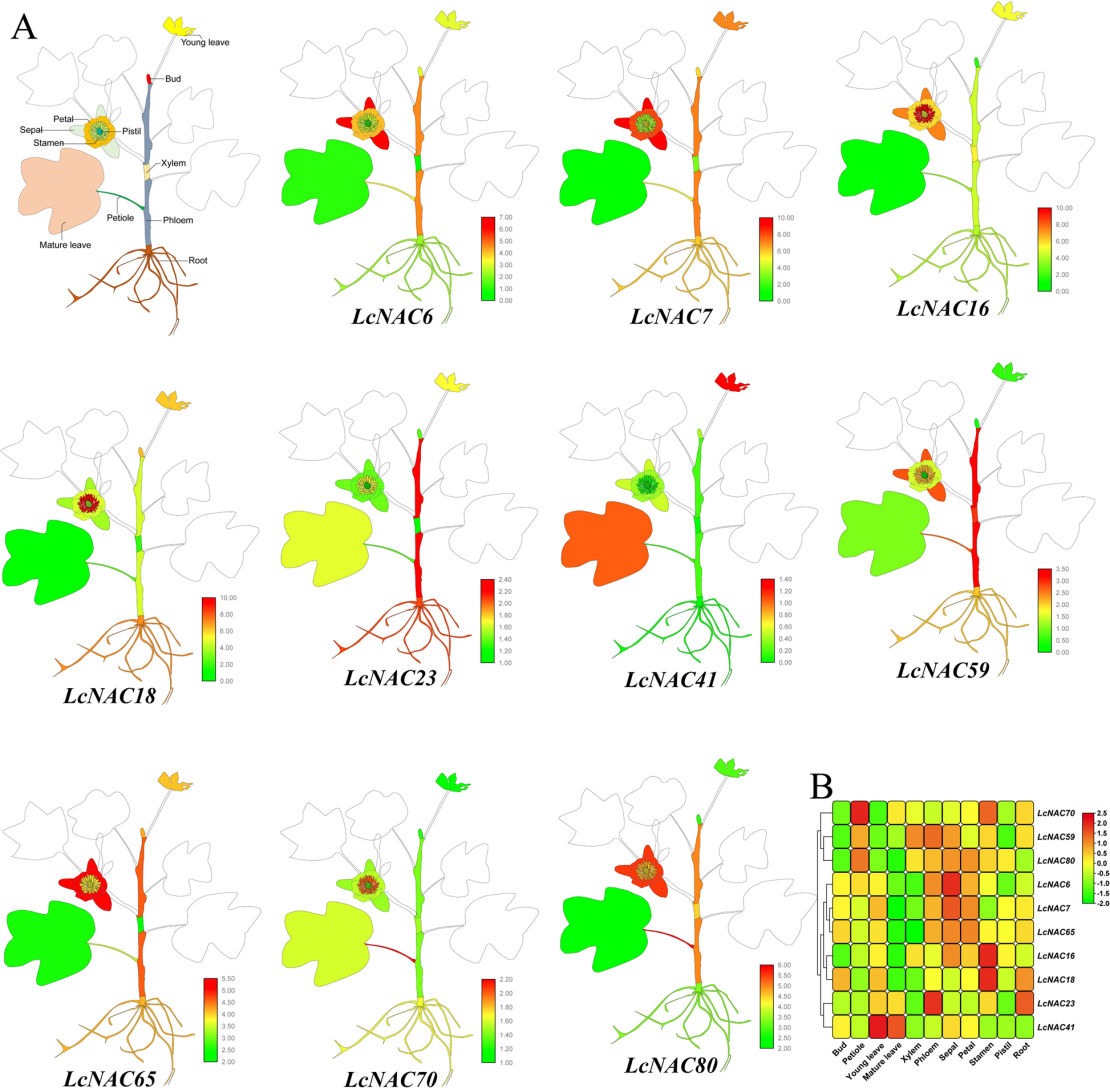
Expression analysis of *LcNAC* genes in different tissues based on RT-qPCR. **A** Diagram showing the different tissues of *L. chinense*. The first figure indicates the annotation of different tissues of the plant, the color is only for the convenience of distinguishing different tissues, not the color of the plant itself. The remaining 10 plants represent the cartoon heat map of different tissues expression, with red representing high level of gene expression and green representing low expression level. **B** The heatmap were drew by TBtools using mean values. Red represents high expression level and green represents low expression level. The expression levels are shown using the log2 transform. Three biological replicates were used

To further investigate *LcNAC* gene function, 10 *NAC* genes were selected for expression level analysis in 11 different tissues, including root, xylem, phloem, petiole, mature leaf, young leaf, bud, petal, sepal, stamen and pistil (Fig. 9). 3 genes (*LcNAC7*, *LcNAC65*, *LcNAC80*) are highly expressed in both petals and sepals and 3 genes (*LcNAC16*, *LcNAC18*, *LcNAC70*) are highly expressed in stamens, suggesting that these 6 genes play a role in the *L. chinense* reproductive organs. Some *LcNAC* genes are highly expressed in the vegetative organs. For example, *LcNAC41* is expressed in both young and mature leaves. *LcNAC23* is expressed especially high in roots and phloem. The genes highly expressed in the xylem are *LcNAC7*, *LcNAC16*, *LcNAC59* and *LcNAC80*.

### Expression analysis of *LcNAC* gene under abiotic stress

**Fig. 10:**
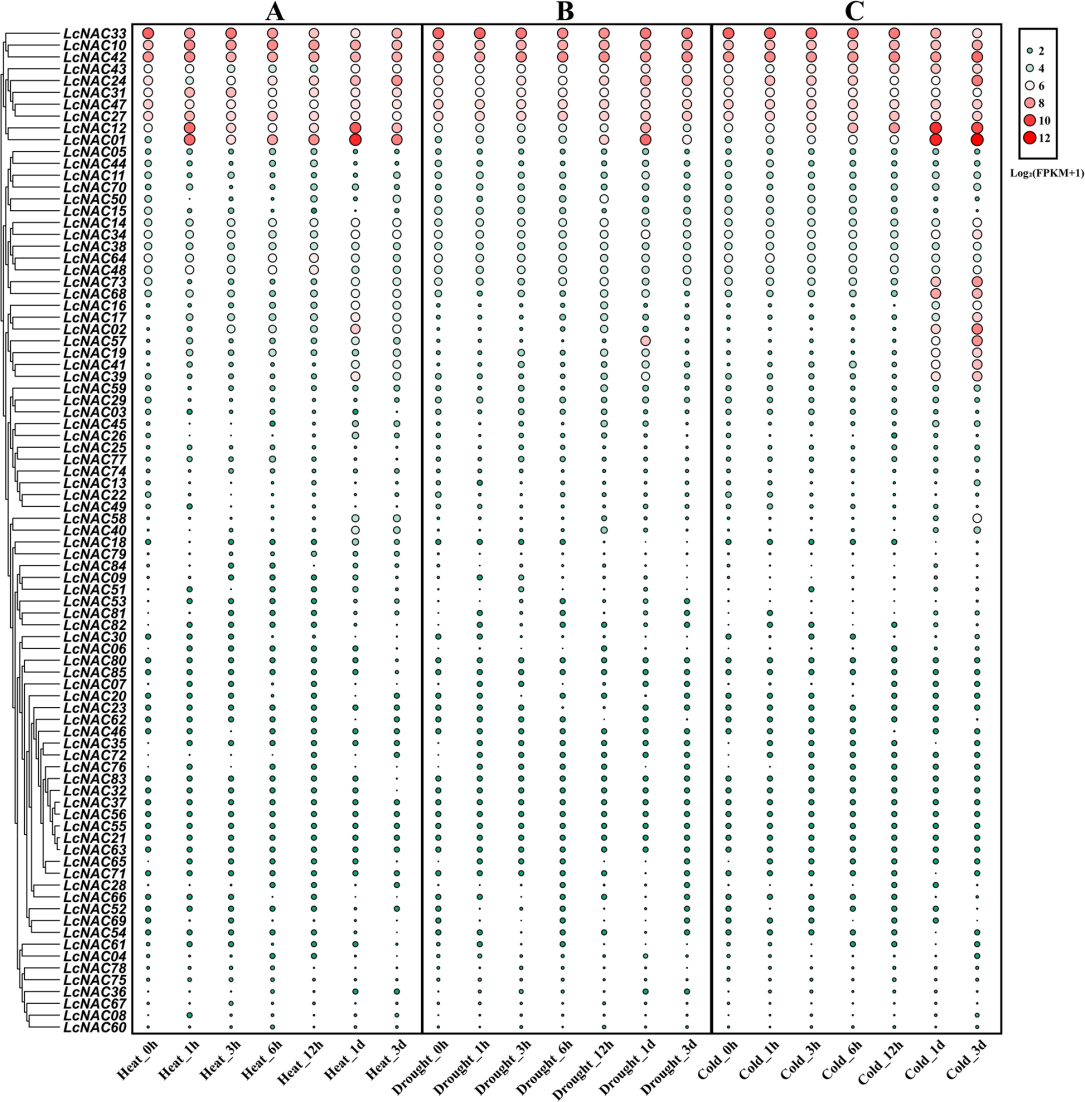
Transcriptional expression patterns of *NAC* genes in *L. chinense* under (**A**) heat, (**B**) drought and (**C**) cold stress. The *LcNACs* were subjected to three different stress factors: Cold-, Heat-, and Drought stress. Heat_0h, Heat_1h, Heat_3h, Heat_6h, Heat_12h, Heat_1d, Heat_3d represent three biological repetitions of each time point (0 h, 1 h, 3 h, 6 h, 12 h, 1d, 3d). Transcript abundance levels are shown using the log2(FPKM + 1) transform. The values on the right panel of the heatmap indicate the level of expression

The *NAC* gene family has been reported to be widely involved in response to abiotic stress [36]. Therefore, we were interested to know whether this is the case for *LcNAC* genes in *L. chinense*. We treated seedlings with three types of abiotic stresses (heat, drought and cold) for 0 h, 1 h, 3 h, 6 h, 12 h, 24 h and 3d. Plant materials were collected, and total RNA was isolated, followed by RNA-Seq analysis. After heat treatment, the expression of 11 *LcNAC* genes is continuously up-regulated during the treatment (Fig. 10). By contrast, the expression of 6 *LcNAC* genes is continuously down-regulated during the treatment. The expression of the other 11 genes decreased first, then increased again. After cold treatment, 15 *LcNAC* genes are continuously up-regulated, while 9 *LcNAC* genes were persistently down-regulated. The expression level of 16 *LcNAC* genes increased first, then decreased only to increase again, in a wave-like manner. After drought treatment, there were 10 *LcNAC* genes with the same trend and double peak expression trend increased twice. Another seven genes went down and then up. Only 6 *LcNAC* genes are consistently up-expressed and 4 *LcNAC* genes were consistently down-expressed (Supplementary Figure S1). These various types of expression indicate that there are likely to be functional differences between *LcNAC* genes in response to abiotic stress.

**Fig. 11:**
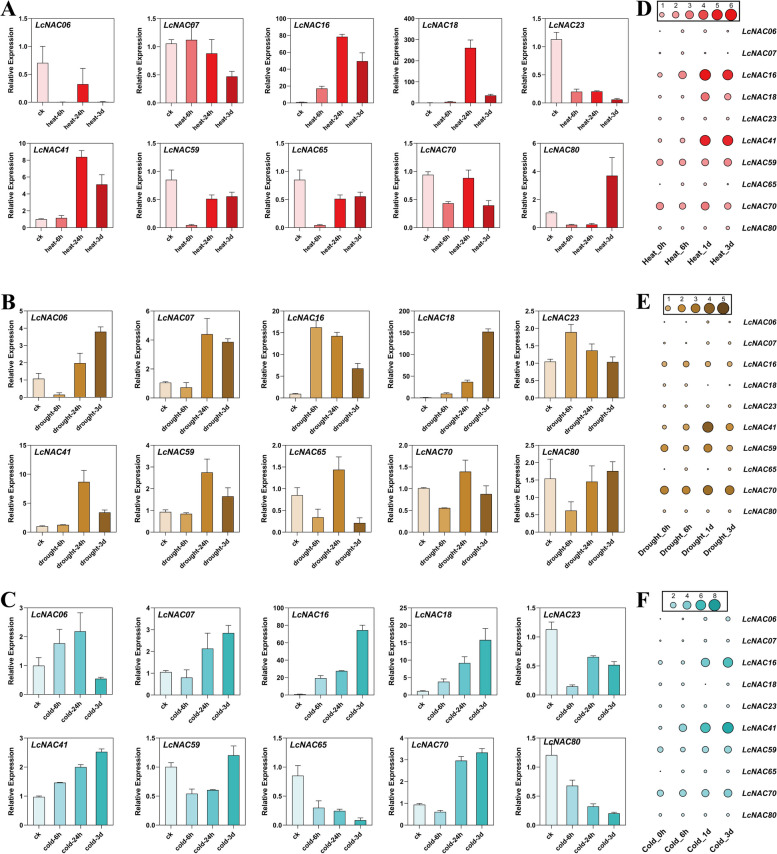
*NAC* gene expression in *L. chinense* under multiple abiotic stresses. **A**-**C** RT-qPCR; **D**-**F** RNA-Seq. The relative mRNA levels are depicted in the y-axis over time in the x-axis. Red, brown, and blue represent heat, drought, and low temperature stresses, respectively. The area of the circle represents the gene expression level. The bar at the top of the heat map indicates the level of expression. The error line of the bar graph represents the mean ± SD (*n* = 3)

To validate our RNA-Seq results, we selected 10 *NAC* genes from the NAM subfamily for RT-qPCR analysis, since the NAM subfamily members is known to be involved in plant growth and development [36]. We harvested the leaves of *L. chinense* that were treated with cold, heat and drought stress for the analysis, and the expression of these *LcNAC* genes are upregulated and downregulated to variable degrees after the treatment. 40% of *LcNAC* genes (*LcNAC06*, *LcNAC23*, *LcNAC59*, *LcNAC65*) were down-regulated after 40℃ high temperature treatment compared with the control group (Fig. 11A). Besides, there were some genes that responded dramatically to high temperature treatment (*LcNAC16*, *LcNAC18*, *LcNAC41*, *LcNAC80*). The representative gene was *LcNAC18*, its expression level did not change significantly during the period of 0-6 h after high temperature treatment, but showed a leap at the time point of 24 h, and was enhanced many times compared with the control group, *LcNAC18* also peaked at the time point of 24 h in the transcriptome (Fig. 11D). The changes in gene expression levels after drought treatment were obviously different from the previous two stresses, and the changes in gene expression trends were more diverse (Fig. 11B). Only *LcNAC18* expression was consistently up-regulated, with trends for most genes fluctuated at the 3-day drought treatment time point. The *LcNAC16* and *LcNAC23* genes increased by 15 and 2 times of the control at 6 h, and the expression trends of these two genes were consistent with those of the RNA-Seq (Fig. 11E). It is possible that osmotic stress due to drought enhanced transcription levels in the short term. In addition, 50% of the genes (*LcNAC07*, *LcNAC41*, *LcNAC59*, *LcNAC65*, *LcNAC70*) expression peaked at 24 h after drought treatment, and 20% of the genes (*LcNAC06*, *LcNAC80*) expressed with peak expression at 3-day after drought treatment. The peak expression of the *LcNAC* gene at different epochs indicated a temporal discrepancy in the response to drought stress in members of the *LcNAC* gene family. The expression levels of three genes (*LcNAC16*, *LcNAC18* and *LcNAC41*) were continuously increased with increasing time after cold treatment (Fig. 11C). On the contrary, the expression levels of two genes (*LcNAC65* and *LcNAC80*) were continuously down-regulated within 3 days of treatment. The RNA-Seq results showed that the expression of *LcNAC16*, *LcNAC18* and *LcNAC41* also increased (Fig. 11F). Overall, our RNA-seq data were consistent with our RT-qPCR results, indicating that our transcriptome data were reproducible.

### Subcellular localization of *LcNAC* genes

**Fig. 12:**
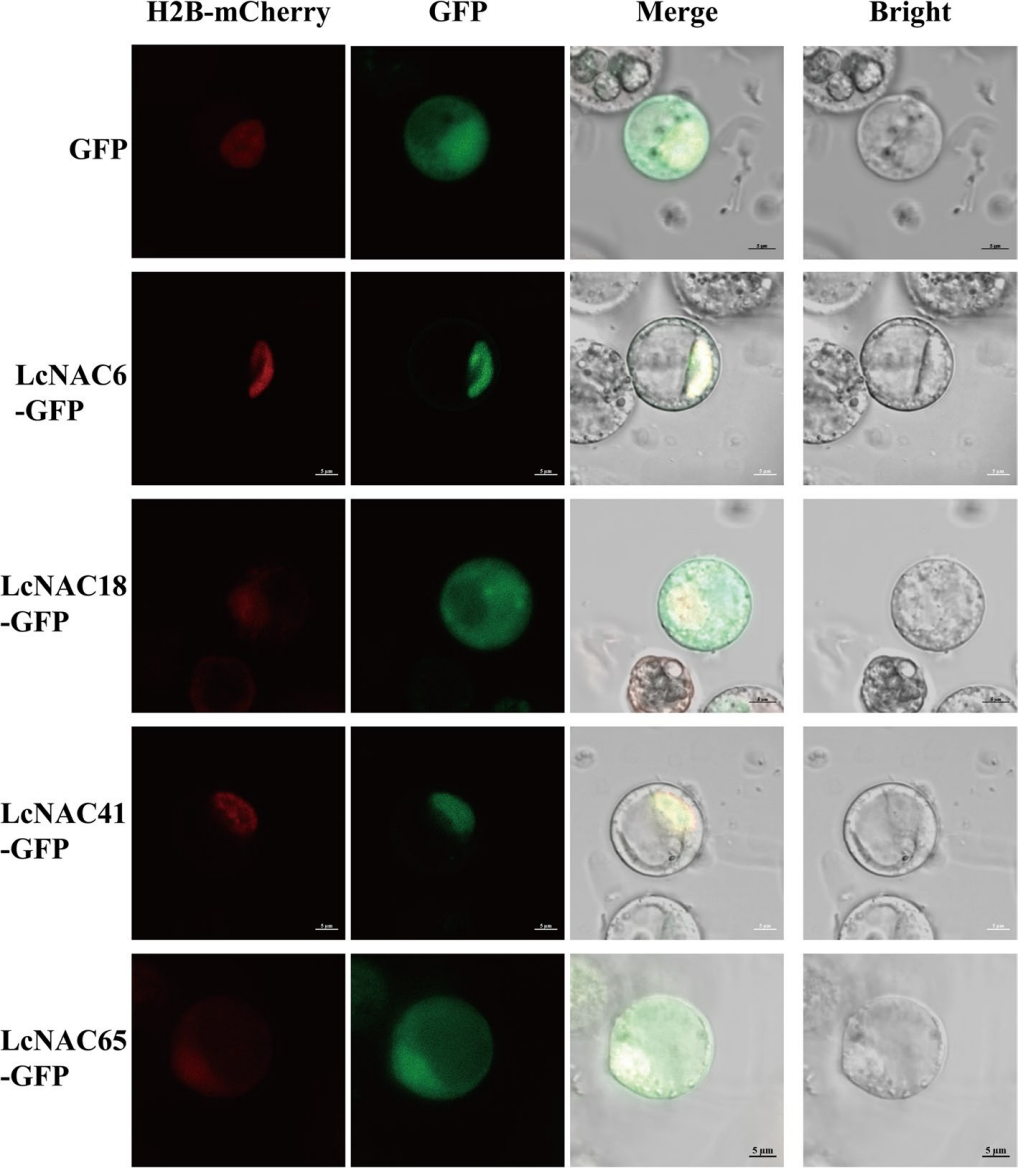
NAC subcellular localization in *L. chinense*. H2B-mCherry (first panel) indicates red fluorescence photography. GFP indicates (second panel) green fluorescence photography. The merged third panel indicates the fusion of red fluorescence, green fluorescence and bright field, the rightmost panel shows the bright field images. Bar = 5 μm

Subcellular localization provides important insight into a protein’s function. In order to determine the subcellular localization of *LcNAC* genes, *LcNAC6, LcNAC18, LcNAC41* and *LcNAC65* were selected for subcellular localization analysis. The plasmids CaMV35S:LcNAC6-GFP, CaMV35S:LcNAC18-GFP CaMV35S:LcNAC41-GFP, CaMV35S:LcNAC65-GFP, CaMV35S:GFP, and CaMV35S:H2B-mCherry were transformed into *L. chinense* protoplasts by PEG mediated transformation (Fig. 12). The signal could be detected in the whole cell when CaMV35S:GFP was transformed. The results showed that CaMV35S:LcNAC6-GFP and CaMV35S:LcNAC41-GFP were typical transcription factors localized only in the nucleus. CaMV35S:LcNAC18-GFP and CaMV35S:LcNAC65-GFP not only emit strong fluorescence signals mainly in the nucleus, but also weak signals are detected in the cytoplasm. It is speculated that these two genes may play a role in cytoplasm in addition to the nuclear regulation of transcription factors, and the specific cytoplasm functions remain to be explored in the next step.

## Discussion

The *NAC* transcription factor family is one of the largest families of transcription factors in plants and has been identified and characterized in a variety of species such as *Medicago truncatula* [37], *Cleistogenes songorica* [38], *Arachis hypogaea* [39], *Musa Acuminata* [40]. The *NAC* gene family in *Liriodendron chinense* remains so far undescribed however. In this study, a total of 85 *NAC* gene family members of *L. chinense* were identified genome wide, localized on 17 chromosomes. *LcNAC* transcription factors could be divided into 16 subfamilies, of which the ANAC063 subfamily contains the most members in *L. chinense*.

In the evolutionary process of many gene families, there is a diversity of gene structure, which is beneficial to create new functions to adapt to environmental changes. The gene structure of *LcNAC* genes is similar to that of *Fagopyrum tataricum* [41]. The N-terminus of *NAC* transcription factors is highly conserved, and members of different subfamilies contain non-identical motifs, but all contain the DNA binding domain.

Increasing the number of genes in a species depends on gene duplication events, which occur at non-regular frequencies across different lineages. A total of 20 fragment duplication genes occurred in *L. chinense*, and *LcNAC* gene duplication occurred on 11 chromosomes (Fig. 4). In addition, the KA/KS value was less than 1, indicating that *LcNAC* genes received purification selection during evolution (Supplementary Table S6). These results are similar to those of previous studies on *L. chinense*. Collinearity analysis showed that there are more orthologous genes between *L. chinense* and *P. trichocarpa* than between *L. chinense* and *A. thaliana*, which may be due to the separate evolution of herbaceous and woody plants. In accordance with the results of previous articles in our laboratory [42], the collinearity between *P. trichocarpa* and other woody plants is stronger, which may be related to the change of the different environment between woody plants and herbaceous plants.

Predicting the regulatory network of unknown proteins from the regulatory network of known proteins is a common method to study the regulatory network of unknown proteins in different species. In this study, using *A. thaliana* proteins as the reference, it was predicted that nine LcNAC proteins play key roles in the protein regulatory network of* L. chinense*. Nine LcNAC proteins have regulatory relationships with other proteins, and the ATAF subfamily plays a key role in the protein regulatory network. In addition, there is an interaction between BABY BOOM (BBM) family related proteins and NAC proteins, which is consistent with previous studies that there is a mutual regulatory relationship between NAC proteins and BBM proteins [43]. There is also an interaction between the xylem development related factor VND1 and NAC proteins. The genes regulating the formation and differentiation of *L. chinense* flower organs also play a key role in the network. The promoters of *LcNACs* mainly contain stress response elements (low temperature, high temperature and drought response elements) and growth and development related elements (light response, auxin response elements), suggesting that *LcNAC* genes are involved in the growth and development of *L. chinense* and the process of coping with abiotic stress.

One way to uncover gene function is to analyze the gene expression pattern. There is obvious diversity in the expression patterns of *LcNAC* genes across the 11 stages of somatic embryogenesis. The members of NAP and NAM subfamilies are significantly higher expressed during the ES3 and ES4 stages of embryogenesis. The results of this study are similar to those of *Akebia trifoliata* (Thunb.) Koidz, both of which indicate that *NAC* family members are involved in somatic embryogenesis, especially in early development [44]. In addition, most of the *LcNAC* genes show tissue specific expression, especially the high expression abundance of NAM subfamily members in the reproductive organs of *L. chinense*, suggesting that they may play an important role in *L. chinense* reproduction.

In this study, RNA-Seq and RT-qPCR were combined to examine the expression patterns of *NAC* gene members in *L. chinense* under three abiotic stresses. The lowest expression of most *LcNAC* genes was at 6 h after low temperature stress, which may be due to the sudden stimulation of low temperature, leading to enzymatic activity in the plant being reduced, the metabolism slowing down, and the defense system not being ready. The expression characteristics of the *LcNAC* genes are similar to those of the homologous gene *SlNAC10* [45]. The gene expression level is generally higher after 24 h of low temperature, compared to 6 h of cold treatment. We speculate that the plant has fully activated their defense system after a period of abiotic stress and enhanced the expression level of genes related to low temperature response through signal transmission, including the expression of *NAC* gene family members. In particular, the expression of *LcNAC18* and *LcNAC65* from the NAM subfamily changed dramatically with the three types of abiotic stress response we applied, suggesting that these two genes play a key role in response to multiple abiotic stresses. Furthermore, most *NAC* transcription factors have been shown to localize to the nucleus, and we found that LcNAC18 and LcNAC65 are present both in the cytoplasm and nucleus, which is not consistent with our predictions.

## Conclusion

This study performed a comprehensive genomic characterization of the *L. chinense* NAC transcription factors. We identified a total of 85 *LcNACs*, distributed across 17 chromosomes. Phylogenetic analysis indicated that LcNACs can be classified into 17 distinct subfamilies. The expression patterns of *LcNAC* genes across different tissues and somatic embryos were explored, and the expression patterns of *LcNAC* genes under abiotic stress were analyzed. Investigation of the expression profiles revealed that *LcNACs* respond to external stimuli (cold, drought and heat stress), among which *LcNAC6/18/41/65* responded to all three stresses investigated. This study provides the basis for further investigating the function of *LcNACs* under abiotic stresses.

### Supplementary Information

**Additional file 1: Table S1.** The Fragments Per Kilobase per million mapped fragments (FPKM) data of NAC genes at different stages of somatic embryos.

**Additional file 2: Table S2.** FPKM values of LcNACs under heat, drought and cold stress.

**Additional file 3: Table S3.** The specific primers used in the qRT-PCR analysis for the selected LcNACs.

**Additional file 4: Table S4.** Characteristics of NAC in L. chinense.

**Additional file 5: Table S5.** The conserved motifs of the genes in the LcNAC gene family are determined by MEME.

**Additional file 6: Table S6. **The Ka/Ks values of LcNAC linked genes.

**Additional file 7: Figure S1.** Clustering analysis of RNA-seq expression trends for LcNAC genes by gene number.

## Data Availability

The datasets used and analyzed during the current study are available in the manuscript and its additional files. The original contributions presented in the study are publicly available. Genome and gene model annotations files are available on the NCBI website (https://www.ncbi.nlm.nih.gov/assembly/GCA_003013855.2). RNA-seq data can be found here: NCBI, accession numbers: PRJNA679089 and PRJNA679101. All materials were obtained with permission, and are available by contacting the corresponding authors (jshi@njfu.edu.cn and chengtl@njfu.edu.cn).
