# Supplementary material for: Genome-wide identification of the NAC gene family and its functional analysis in Liriodendron
Source: BMC Plant Biol. 2023 Sep 8;23:415. doi: 10.1186/s12870-023-04415-4 (PMC10486064; doi:10.1186/s12870-023-04415-4)
Supplement: Supplementary file 7 — Additional file 7: Figure S1. Clustering analysis of RNA-seq expression trends for LcNAC genes by gene number. [file 12870_2023_4415_MOESM7_ESM.docx]

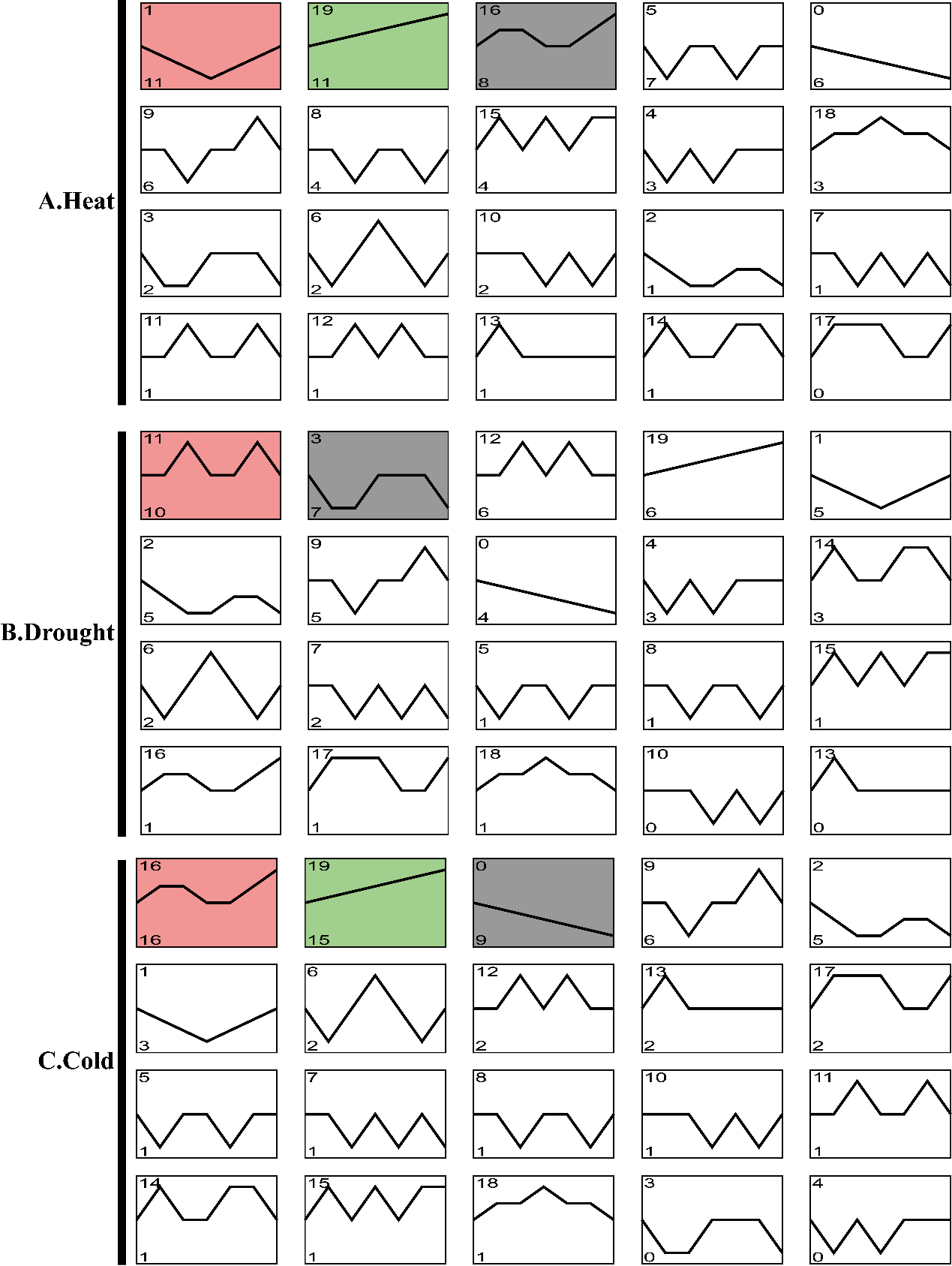


**Figure S1.** Clustering analysis of RNA-seq expression trends for *LcNAC* genes by gene number.

Note: The top-left value indicates the ID of the trend and the bottom-left value indicates the number of genes in the trend. Colored trend block: a significant enrichment trend. The different colors are set by the software to distinguish different trends, and the colors have no special meaning. Trend block without color: non-significant enrichment trends
